# Content of macronutrients in oat (*Avena sativa* L*.*) after remediation of soil polluted with cobalt

**DOI:** 10.1007/s10661-019-7529-6

**Published:** 2019-05-22

**Authors:** Milena Kosiorek, Mirosław Wyszkowski

**Affiliations:** Department of Environmental Chemistry, University of Warmia and Mazury in Olsztyn, Plac Łódzki 4, 10-727 Olsztyn, Poland

**Keywords:** Cobalt, Soil amendments, *Avena sativa* L., Macroelements

## Abstract

The purpose of this study was to determine the remediation effect of various substances (manure, clay, charcoal, zeolite and calcium oxide) on limiting the influence of high cobalt doses (0, 20, 40, 80, 160 and 320 mg/kg of soil) on the content of macroelements in grain, straw and roots of oat. The doses of cobalt applied in this experiment as well as soil amendments such as manure, clay, charcoal, zeolite and calcium oxide had a significant effect on the content of the analysed macronutrients in grain, straw and roots of oat. In the series without any neutralising substances, the soil contamination with cobalt caused an increase in the content of nitrogen, phosphorus, sodium, calcium and, partly, potassium, in grain, straw and roots of oat. Among the neutralising substances tested, the most unambiguous effect was produced by manure, which raised the content of all macronutrients (except calcium and magnesium) in oat grain, straw and roots. The influence of the other substances on the content of macronutrients in oat plants was less equivocal. However, all of them, especially calcium oxide, tended to induce a decrease in the content of most macronutrients in grain, straw and roots of oat.

## Introduction

Cobalt is one of the metals composing the Earth’s crust. Its highest concentrations are found in sulphate and arsenate ores as well as in oxide minerals of such metals as manganese, copper and nickel. Globally, the average cobalt content in soils is 8 mg/kg (Bowen 1979). In Poland, the content of cobalt in soils does not exceed its allowable amount set for arable land (group II, sub-group of soils II-1), which equals 20 mg/kg, as stipulated in the Regulation of the Minister of the Environment of 1 September 2016 on the conduct of the assessment of contamination of the surface of the earth (Toxicological Profile for Cobalt 2004). Beyond the borders of our country, there are sites where the content of this metal in soil significantly exceeds its permissible levels. The amounts of cobalt in soils across Europe are within the range of 0.5 to 255 mg/kg (Cappuyns and Mallaerts 2014). The strongest impact on its content is played by natural factors, mostly the type of rock from which given soil developed, but also by human action, due to the widespread use of cobalt in various branches of industry (Adriano 2001). As well as being inhabited by many organisms, soil participates in the flow of energy and matter, and it plays an important role in biomass production (Bouma 2014).

The presence of adequate amounts of nutrients in soil is essential for the development of any crop plant. The intensity of nutrient uptake is much higher during the initial stages of plant development. At that time, the highest accumulation of nutrients occurs in vegetative parts of plants. In the subsequent growth stages, the demand for micro- and macronutrients decreases, and the nutrients absorbed by plants accumulate in storage parts (Imran and Gurmani 2011). The uptake of cobalt by plant roots (most often in the form of Co^2+^ or as chelates) occurs via transfer through cell membranes, and further distribution may also involve organic complexes. The mobility of cobalt in a plant is low and in particular is limited during its transfer from roots to aerial parts (Barysas et al. 2002; Bakkaus et al. 2005).

Plants tend to take up small amounts of cobalt. The average content of cobalt in plants oscillates between 0.01 and 1 mg/kg of dry matter (Walsh 1971). An amount of cobalt in excess of its physiological content in plant tissues can induce the occurrence of harmful pathological signs and may have a disturbing influence on the uptake of other macro- and micronutrients (Hemantaranjan et al. 2000). The strongest effect on the uptake of this element is attributed to the plant’s species, age and mechanisms which occur in the plant. A significant role is also played by soil properties and climatic conditions characteristic for a given territory (Kashin 2011; Kosiorek and Wyszkowski 2016a, b). It is therefore essential to search for optimal methods to limit cobalt uptake by plants, for example through application of different substances to soil and by using phytoremediation abilities of plants (Pszczółkowski et al. 2012).

Considering the above, a study was undertaken with the purpose to determine the effect of various substances (manure, clay, charcoal, zeolite and calcium oxide) on limiting the influence of high cobalt doses on the content of macroelements in grain, straw and roots of oat.

## Material and methods

### Methodological approach

The research was based on a pot experiment conducted in a greenhouse at the University of Warmia and Mazury in Olsztyn (north-eastern Poland). Polyethylene pots, each holding 9 kg of soil, were used to set up the trials. The experiment was conducted with three replications, on loamy sand with the following shares of fractions: sand (> 0.05 mm) 73.9%, silt (0.02–0.05 mm) 24.1% and clay (< 0.002 mm) 2.0%. The soil had the following characteristics: pH in 1 M KCl—5.05, hydrolytic acidity—28.40 mmol(+)/kg, total exchangeable bases—46.50 mmol(+)/kg, cation exchange capacity—74.90 mmol(+)/kg, base saturation of the sorption complex—68.08%, content of organic carbon—12.15 g/kg, content of available forms of phosphorus—40.99 mg/kg, potassium—46.29 mg/kg and magnesium—116.21 mg/kg, content of total cobalt—9.98 mg/kg of soil. The soil was polluted with increasing doses of cobalt: 0, 20, 40, 80, 160, 320 mg/kg of soil (cobalt chloride) and mixed with substances such as granulated bovine manure, clay, charcoal, zeolite (in an amount equal 2% of soil mass per pot) and calcium oxide (in a dose corresponding to 1 hydrolytic acidity). Source of origin of the tested substances was as follows: granulated bovine manure (granulation diameter from 4 to 5 mm) from the Fertigo Company, clay (0.002 mm fraction) from the subsoil layer (30–60 cm deep into the soil) in Tomaszkowo, near Olsztyn, Poland (53° 42′ 50.73″ N, 20° 26′ 02.41″ E), charcoal—hardwood species of European origin from Dancook Company, zeolite (clinoptilolite type)—originating from Central Mexico’s zeolite deposits (Villa de Reyes, San Luis Potosi State) from EREM Company, calcium oxide—Kujawit type fertilizer from ANKOM Kielce Company. The chemical composition of these substances was as follows: granulated bovine manure: Co—0.529 mg/kg, P—21.80 g/kg, K—16.30 g/kg, Na—0.379 g/kg, Ca—28.61 g/kg, Mg—6.020 g/kg; clay: Co—2.956 mg/kg, P—1.088 g/kg, K—1.290 g/kg, Na—0.161 g/kg, Ca—14.48 g/kg, Mg—0.525 g/kg; charcoal: Co—0.200 mg/kg, P—0.260 g/kg, K—0.766 g/kg, Na—0.172 g/kg, Ca—15.01 g/kg, Mg—0.477 g/kg; zeolite: Co—0.313 mg/kg, P—1.836 g/kg, K—5.435 g/kg, Na—0.215 g/kg, Ca—15.85 g/kg, Mg—0.553 g/kg; calcium oxide: Co—1.632 mg/kg, P—0.088 g/kg, K—0.436 g/kg, Na—0.073 g/kg, Ca—351.87 g/kg, Mg—0.901 g/kg. The choice of the above cobalt doses was made in accordance with permissible concentrations of this metal as set in the Regulation of the Minister of the Environment, of 9 September 2002, on the soil quality standards and the earth quality standards (the currently binding legal act is the Regulation of the Minister of the Environment of 1 September 2016 on the assessment of the land surface contamination). Doses of soil amendments were determined based on their beneficial effect on soil physicochemical properties. In addition, each pot was once treated with 100 mg N (NH_4_NO_3_), 35 mg P (KH_2_PO_4_), 100 mg K (KH_2_PO_4_ + KCl), 50 mg Mg (MgSO_4_·7H_2_O), 0.33 mg B (H_3_BO_3_), 5 mg Mn (MnCl_2_·4H_2_O) and 5 mg Mo ((NH_4_)_6_Mo_7_O_24_·4H_2_O)/kg of soil. The doses of cobalt, soil amendments and fertilising nutrients were applied to soil at the onset of the experiment. Afterwards, each pot was sown with oat (*Avena sativa* L.) of the variety Zuch, at a plant density of 15 plants per pot. Oat was harvested after 78 days, at full ripeness stage, and the harvested plants were separated into grain, straw and roots.

### Methods of laboratory and statistical analyses

Prior to chemical analyses, the plant material was dried in adequately prepared dryers, at a temperature of 60 °C, and subsequently ground in an electric mill. The plant material thus prepared underwent ‘wet’ mineralisation in concentrated 95% sulphuric acid, after which the following determinations were made: total nitrogen by the Kjeldahl’s method (Bremner 1965), phosphorus by the colorimetric method (Ostrowska et al. 1991), and potassium, calcium, magnesium and sodium by atomic absorption spectrometry (Ostrowska et al. 1991). The results were analysed statistically with the help of two-factorial analysis of variance ANOVA and PCA, supported by Statistica software (Dell Inc. 2016).

## Results

The application of increasing doses of cobalt as well as various types of substances neutralising the impact of this metal on soil, such as manure, clay, charcoal, zeolite and calcium oxide, had a significant effect on the content of analysed macronutrients in oat grain, straw and roots.

### Nitrogen

In the series without neutralising substances, in treatments with 20, 40 and 80 mg Co/kg of soil, a slight increase in total nitrogen was noted in oat grain, while its rise in straw (by 90%) and roots (by 56%) was significant (Table 1). In soil contaminated with the highest cobalt doses, the growth and development of the test plant was either completely inhibited or very severely retarded, which prohibited an analysis of the plant material with respect to its content of macronutrients. When charcoal, zeolite and calcium oxide had been added to soil, the content of nitrogen decreased in all organs of oat, although the strongest impact was observed to have been produced by calcium oxide on grain, straw and roots of oat. These substances caused a decrease in total nitrogen by 8, 10 and 13%, respectively, to the plant organs mentioned, in comparison to the series without added neutralising substances. The application of manure had the strongest effect on the increase in the amount of nitrogen in oat straw (by 9%) and roots (by 8%).

**Table 1:** Content of nitrogen in oats grain, straw and roots

Dose of cobalt (mg/kg of soil)	Without amendments	Kind of amendments					
		Manure	Clay	Charcoal	Zeolite	Calcium oxide	Average
Grain (g/kg)							
0	27.25	27.97	28.39	28.91	27.48	26.58	27.76
20	28.74	29.35	29.26	28.84	27.67	27.06	28.49
40	28.98	29.12	29.38	27.39	28.07	26.44	28.23
80	28.42	30.31	27.13	21.93	27.16	26.16	26.85
160	n.a.	28.28	22.44	21.91	22.98	24.20	23.96
320	n.a.	23.49	n.a.	n.a.	n.a.	n.a.	23.49
Average	28.35	28.09	27.32	25.80	26.67	26.09	26.46
*r*	0.496*	− 0.828**	− 0.929**	− 0.888**	− 0.900**	− 0.940**	− 0.915**
LSD	a 0.760**, b 0.760**, a·b 1.861**						
Straw (g/kg)							
0	15.52	19.85	18.97	18.13	19.57	19.37	18.57
20	17.33	19.88	18.73	19.08	19.01	19.22	18.88
40	17.66	20.91	19.53	17.31	18.48	19.79	18.95
80	19.34	24.06	19.36	16.28	19.69	17.62	19.39
160	29.26	26.15	27.18	24.29	25.97	17.19	25.01
320	29.42	28.86	33.30	26.99	n.a.	22.44	28.20
Average	21.42	23.29	22.85	20.35	20.54	19.27	21.50
*r*	0.908**	0.959**	0.974**	0.886**	0.884**	0.507*	0.969**
LSD	a 0.858**, b 0.858**, a·b 2.102**						
Roots (g/kg)							
0	14.19	17.54	14.77	15.44	13.95	14.39	15.05
20	17.64	17.13	15.89	15.33	14.00	15.42	15.90
40	14.93	17.61	15.00	15.66	14.74	15.98	15.65
80	16.73	17.62	15.91	15.12	15.21	14.46	15.84
160	22.14	19.51	21.58	18.36	20.14	14.63	19.39
320	n.a.	21.77	n.a.	n.a.	n.a.	n.a.	21.77
Average	17.13	18.53	16.63	15.98	15.61	14.98	17.27
*r*	0.877**	0.979**	0.919**	0.840**	0.957**	− 0.271	0.973**
LSD	a 0.580**, b 0.580**, a·b 1.422**						

LSD for: a—cobalt dose, b—kind of amendment, a·b—interaction; significant at **P = 0.01, *P = 0.05; *r*—correlation coefficient; *n.a*.—not analysed

### Phosphorus

In the series without neutralising substances, the content of phosphorus increased by 15% in grain, by 86% in straw and by 19% in roots of oat exposed to the soil contamination with cobalt (Table 2). The application of manure to soil had the strongest impact on an average increase in the content of this macronutrient in grain (by 11%), straw (by 85%) and roots (by 18%). The remaining substances caused a decrease in the content of phosphorus in oat grain and roots, and their influence on root phosphorus content was much stronger than on grain. A decrease in the content of phosphorus in oat roots ranged from 19% (zeolite) to 38% (calcium oxide). Calcium oxide also caused a 12% decrease, while zeolite and clay were responsible for a 11–12% increase in the content of phosphorus in straw.

**Table 2:** Content of phosphorus in oats grain, straw and roots

Dose of cobalt (mg/kg of soil)	Without amendments	Kind of amendments					
		Manure	Clay	Charcoal	Zeolite	Calcium oxide	Average
Grain (g/kg)							
0	2.506	2.814	2.637	2.552	2.496	2.393	2.566
20	2.631	3.085	2.528	2.623	2.514	2.389	2.628
40	2.636	3.035	2.496	2.570	2.576	2.457	2.628
80	2.888	3.046	2.548	2.589	2.595	2.375	2.674
160	n.a.	2.931	2.190	2.263	2.225	2.643	2.450
320	n.a.	2.765	n.a.	n.a.	n.a.	n.a.	2.765
Average	2.665	2.946	2.480	2.519	2.481	2.451	2.619
*r*	0.972**	− 0.577**	− 0.912**	− 0.851**	− 0.728**	0.833**	0.392**
LSD	a 0.067**, b 0.067**, a·b 0.163**						
Straw (g/kg)							
0	1.033	2.510	1.235	1.226	1.339	1.154	1.416
20	1.196	2.524	1.131	1.094	1.353	1.103	1.400
40	1.027	2.684	1.127	0.911	1.440	1.026	1.369
80	1.012	2.625	1.309	0.934	1.205	1.110	1.366
160	1.851	2.167	1.998	1.681	2.130	1.107	1.822
320	1.919	2.373	2.237	1.838	n.a.	1.553	1.984
Average	1.340	2.481	1.506	1.281	1.493	1.176	1.560
*r*	0.876**	− 0.569**	0.941**	0.834**	0.798**	0.853**	0.928**
LSD	a 0.138**, b 0.138**, a·b 0.339**						
Roots (g/kg)							
0	2.144	2.759	1.668	1.581	1.820	1.497	1.912
20	2.339	2.663	1.555	1.770	1.981	1.356	1.944
40	1.957	2.610	1.528	1.659	1.654	1.255	1.777
80	2.149	2.652	1.806	1.659	1.604	1.405	1.879
160	2.555	2.532	2.156	1.700	1.983	1.377	2.051
320	n.a.	2.555	n.a.	n.a.	n.a.	n.a.	2.555
Average	2.229	2.629	1.743	1.674	1.808	1.378	2.020
r	0.647**	− 0.751**	0.910**	0.249	0.197	− 0.152	0.924**
LSD	a 0.081**, b 0.081**, a·b 0.199**						

LSD for: a—cobalt dose, b—kind of amendment, a·b—interaction; significant at **P = 0.01, *P = 0.05; *r*—correlation coefficient; *n.a*.—not analysed

### Potassium

The highest increase in the grain content of potassium (by 21%) was noted in the series without neutralising substances polluted with 40 mg Co/kg of soil, whereas the dose of 160 mg Co/kg of soil resulted in the highest increase in potassium content (by 24%) found in roots of the test plant (Table 3). Reverse correlations were observed regarding straw, but the strongest effect was produced by the dose of 320 mg Co/kg of soil, which resulted in a 32% decrease in the potassium content. Similarly to phosphorus, an average content of potassium in grain, straw and roots of oat grown in soil treated with manure increased by 12, 5 and 16%, respectively. The application of zeolite lowered the content of potassium in all organs of oat while calcium oxide added to soil caused a reduction of potassium in oat grain and straw. Noteworthy, both these substances had a stronger impact on the potassium content in straw than in grain or roots of oat. Moreover, clay and calcium oxide and especially charcoal led to an increase in the potassium content in oat roots. Clay had a contrary effect on the potassium content in oat roots.

**Table 3:** Content of potassium in oats grain, straw and roots

Dose of cobalt (mg/kg of soil)	Without amendments	Kind of amendments					
		Manure	Clay	Charcoal	Zeolite	Calcium oxide	Average
Grain (g/kg)							
0	7.06	8.82	7.57	6.83	7.16	6.85	7.38
20	7.32	8.35	7.37	7.65	7.07	6.80	7.43
40	8.55	9.17	8.07	7.62	7.70	6.63	7.96
80	8.16	9.21	7.15	7.53	7.18	6.59	7.64
160	n.a.	8.61	8.37	7.58	7.56	6.72	7.77
320	n.a.	8.18	n.a.	n.a.	n.a.	n.a.	8.18
Average	7.77	8.72	7.71	7.44	7.33	6.72	7.72
*r*	0.740**	− 0.561*	0.546*	0.463*	0.472*	− 0.425	0.801**
LSD	a 0.155**, b 0.155**, a·b 0.381**						
Straw (g/kg)							
0	21.60	20.31	18.75	21.61	15.10	16.85	19.04
20	21.55	20.94	20.30	21.26	15.36	18.20	19.60
40	21.08	20.10	19.52	20.88	14.05	16.76	18.73
80	19.21	20.50	16.23	17.69	12.65	16.37	17.11
160	15.62	19.56	13.84	14.49	10.46	13.65	14.60
320	14.76	18.15	14.62	14.77	n.a.	11.53	14.77
Average	18.97	19.93	17.21	18.45	13.52	15.56	17.31
*r*	− 0.937**	− 0.938**	− 0.798**	− 0.873**	− 0.984**	− 0.961**	− 0.878**
LSD	a 0.993**, b 0.993**, a·b 2.432**						
Roots (g/kg)							
0	11.01	12.54	12.88	15.42	10.66	14.12	12.77
20	11.25	13.34	11.73	16.06	11.17	14.20	12.96
40	10.84	13.23	11.23	15.49	10.97	14.53	12.72
80	10.75	13.49	13.72	13.77	10.14	13.98	12.64
160	13.70	13.04	14.37	13.99	9.270	12.48	12.81
320	n.a.	14.52	n.a.	n.a.	n.a.	n.a.	14.52
Average	11.51	13.36	12.79	14.95	10.44	13.86	13.07
*r*	0.822**	0.823**	0.735**	− 0.797**	− 0.913**	− 0.882**	0.861**
LSD	a 0.538**, b 0.538**, a·b 1.318**						

LSD for: a—cobalt dose, b—kind of amendment, a·b—interaction; significant at **P = 0.01, *P = 0.05; *r*—correlation coefficient; *n.a*.—not analysed

### Sodium

In treatments without neutralising substances, the most severe impact of cobalt contamination on the sodium content (an increase by 22%) was observed in oat straw under the influence of the dose 80 mg Co/kg of soil (Table 4). Soil remediation with manure and zeolite caused an increase in the sodium content in all analysed oat parts, while the other substances led to the same result but only in oat straw. The effect of the applied substances on the sodium content in straw was much stronger than in grain or roots of oat.

**Table 4:** Content of sodium in oats grain, straw and roots

Dose of cobalt (mg/kg of soil)	Without amendments	Kind of amendments					
		Manure	Clay	Charcoal	Zeolite	Calcium oxide	Average
Grain (g/kg)							
0	0.469	0.544	0.478	0.459	0.500	0.456	0.484
20	0.486	0.545	0.494	0.498	0.517	0.469	0.502
40	0.499	0.606	0.491	0.501	0.572	0.476	0.524
80	0.483	0.608	0.497	0.501	0.538	0.478	0.518
160	n.a.	0.606	0.517	0.505	0.574	0.481	0.537
320	n.a.	0.551	n.a.	n.a.	n.a.	n.a.	0.551
Average	0.484	0.577	0.495	0.493	0.540	0.472	0.519
*r*	0.416	− 0.024	0.949**	0.631**	0.727**	0.800**	0.880**
LSD	a 0.014**, b 0.014**, a·b 0.033**						
Straw (g/kg)							
0	0.495	1.225	0.597	0.573	1.355	0.607	0.809
20	0.485	1.211	0.597	0.547	1.393	0.580	0.802
40	0.512	1.234	0.615	0.593	1.537	0.560	0.842
80	0.605	1.332	0.621	0.708	1.592	0.630	0.915
160	0.540	1.429	0.693	0.726	1.532	0.635	0.926
320	0.561	1.446	0.702	0.777	n.a.	0.635	0.824
Average	0.533	1.313	0.638	0.654	1.482	0.608	0.853
*r*	0.517*	0.905**	0.927**	0.888**	0.669**	0.647**	0.184
LSD	a 0.012**, b 0.012**, a·b 0.029**						
Roots (g/kg)							
0	1.112	1.253	1.036	1.116	1.383	1.016	1.153
20	1.140	1.312	1.030	1.010	1.395	1.028	1.153
40	1.046	1.287	1.049	1.078	1.397	1.050	1.151
80	1.145	1.311	1.058	1.086	1.430	1.101	1.189
160	1.176	1.342	1.137	1.233	1.541	1.147	1.263
320	n.a.	1.460	n.a.	n.a.	n.a.	n.a.	1.460
Average	1.124	1.328	1.062	1.105	1.429	1.068	1.228
*r*	0.585**	0.966**	0.960**	0.780**	0.973**	0.986**	0.986**
LSD	a 0.034**, b 0.034**, a·b 0.084**						

LSD for: a—cobalt dose, b—kind of amendment, a·b—interaction; significant at **P = 0.01, *P = 0.05; *r*—correlation coefficient; *n.a*.—not analysed

### Calcium

The content of calcium in oat grown without neutralising substances added to soil was much higher under the influence of cobalt contamination (by 97% in grain, 28% in straw and 40% in roots of oat) compared to the object without cobalt (Table 5). In the treatments with calcium oxide, higher concentrations of sodium were determined in oat grain and roots, 40% and 14%, respectively. Manure led to a reduction in the content of calcium in all oat organs. Clay, charcoal and zeolite acted analogously with respect to the calcium content in oat straw and root, unlike in grain of oat.

**Table 5:** Content of calcium in oats grain, straw and roots

Dose of cobalt (mg/kg of soil)	Without amendments	Kind of amendments					
		Manure	Clay	Charcoal	Zeolite	Calcium oxide	Average
Grain (g/kg)							
0	2.220	1.660	2.350	2.780	2.520	3.420	2.492
20	2.120	1.810	2.750	3.010	2.360	3.390	2.573
40	2.570	1.800	3.790	3.110	2.830	3.280	2.897
80	4.370	2.000	3.980	3.760	2.980	4.150	3.540
160	n.a.	3.670	3.940	5.890	7.170	5.560	5.246
320	n.a.	4.420	n.a.	n.a.	n.a.	n.a.	4.420
Average	2.820	2.560	3.362	3.710	3.572	3.960	3.528
*r*	0.928**	0.962**	0.766**	0.978**	0.925**	0.959**	0.776**
LSD	a 0.251**, b 0.251**, a·b 0.615**						
Straw (g/kg)							
0	13.910	8.120	11.160	10.300	9.070	11.870	10.738
20	14.990	8.490	11.850	10.350	9.460	12.570	11.285
40	14.630	8.560	12.110	12.250	10.590	12.520	11.777
80	13.830	8.570	12.790	12.990	12.570	13.400	12.358
160	15.950	10.660	14.380	14.050	13.520	15.390	13.992
320	17.830	13.980	17.290	15.850	n.a.	16.150	16.220
Average	15.190	9.730	13.260	12.630	11.04	13.650	12.728
*r*	0.916**	0.985**	0.999**	0.944**	0.955**	0.953**	0.995**
LSD	a 0.837**, b 0,837**, a·b 2.050**						
Roots (g/kg)							
0	4.850	5.410	3.620	4.600	3.790	5.080	4.558
20	4.130	3.980	3.840	4.250	3.810	5.890	4.317
40	4.610	4.130	3.940	4.720	3.810	5.740	4.492
80	4.850	3.350	3.590	5.040	4.810	5.870	4.585
160	6.780	4.710	5.430	5.370	4.870	6.260	5.570
320	n.a.	5.490	n.a.	n.a.	n.a.	n.a.	5.490
Average	5.044	4.512	4.084	4.796	4.218	5.768	4.835
*r*	0.883**	0.444	0.852**	0.900**	0.885**	0.810**	0.860**
LSD	a 0.118**, b 0.118**, a·b 0.288**						

LSD for: a—cobalt dose, b—kind of amendment, a·b—interaction; significant at **P = 0.01, *P = 0.05; *r*—correlation coefficient; *n.a*.—not analysed

### Magnesium

Both increasing doses of cobalt in the series without neutralising substances and the applied substances had little effect on the magnesium content in oat grain, straw and roots (Table 6).

**Table 6:** Content of magnesium in oats grain, straw and roots

Dose of cobalt (mg/kg of soil)	Without amendments	Kind of amendments					
		Manure	Clay	Charcoal	Zeolite	Calcium oxide	Average
Grain (g/kg)							
0	4.354	4.436	4.387	4.423	4.395	4.388	4.397
20	4.345	4.456	4.390	4.426	4.397	4.378	4.399
40	4.375	4.448	4.414	4.422	4.425	4.376	4.410
80	4.448	4.474	4.443	4.458	4.415	4.388	4.438
160	n.a.	4.588	4.412	4.456	4.525	4.388	4.474
320	n.a.	4.492	n.a.	n.a.	n.a.	n.a.	4.492
Average	4.381	4.482	4.409	4.437	4.431	4.384	4.435
r	0.934**	0.523*	0.519*	0.841**	0.935**	0.417	0.948**
LSD	a 0.010**, b 0.010**, a·b 0.025**						
Straw (g/kg)							
0	4.534	4.619	4.577	4.523	4.545	4.573	4.562
20	4.543	4.621	4.588	4.581	4.550	4.604	4.581
40	4.554	4.625	4.595	4.595	4.570	4.629	4.595
80	4.583	4.628	4.598	4.632	4.626	4.651	4.620
160	4.694	4.695	4.677	4.724	4.710	4.711	4.702
320	4.752	4.845	4.848	4.795	n.a.	4.801	4.808
Average	4.610	4.672	4.647	4.642	4.600	4.662	4.645
*r*	0.972**	0.976**	0.982**	0.968**	0.994**	0.990**	0.997**
LSD	a 0.016**, b 0.016**, a·b 0.039**						
Roots (g/kg)							
0	4.369	4.446	4.243	4.185	4.165	4.188	4.266
20	4.366	4.373	4.197	4.232	4.193	4.227	4.265
40	4.387	4.395	4.222	4.224	4.187	4.246	4.277
80	4.393	4.392	4.249	4.255	4.291	4.276	4.309
160	4.634	4.436	4.456	4.381	4.443	4.317	4.445
320	n.a.	4.571	n.a.	n.a.	n.a.	n.a.	4.571
Average	4.430	4.436	4.273	4.255	4.256	4.251	4.355
*r*	0.921**	0.857**	0.899**	0.972**	0.986**	0.966**	0.987**
LSD	a 0.008**, b 0.008**, a·b 0.020**						

LSD for: a—cobalt dose, b—kind of amendment, a·b—interaction; significant at **P = 0.01, *P = 0.05; *r*—correlation coefficient; *n.a*.—not analysed

### PCA and correlation coefficients

**Fig. 1:**
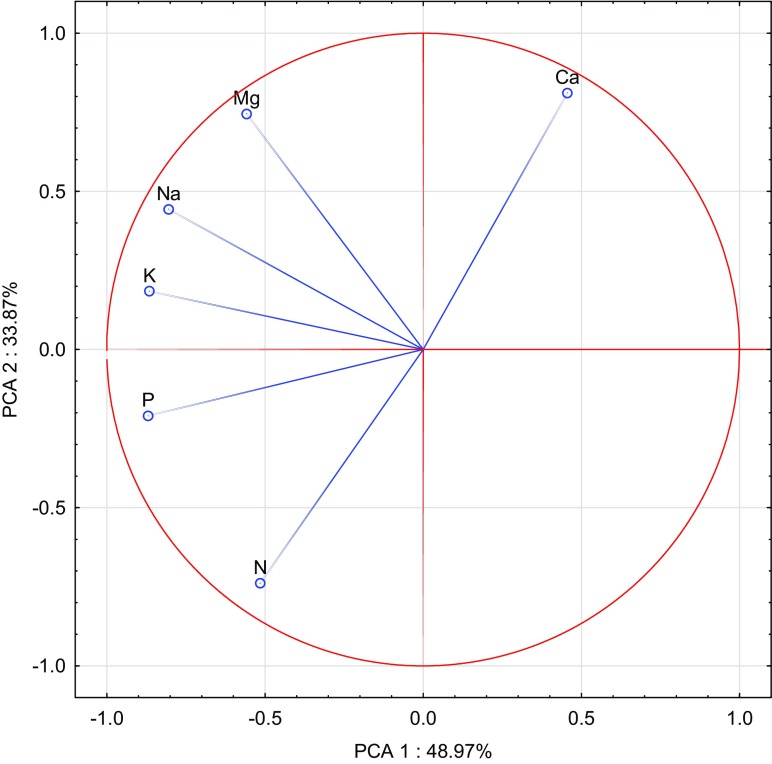
Content of macroelements in the oats grain illustrated with the PCA method. Key: vectors represent analysed variable (content of N, P, K, Na, Ca and Mg) and points show the grain samples with elements

**Fig. 2:**
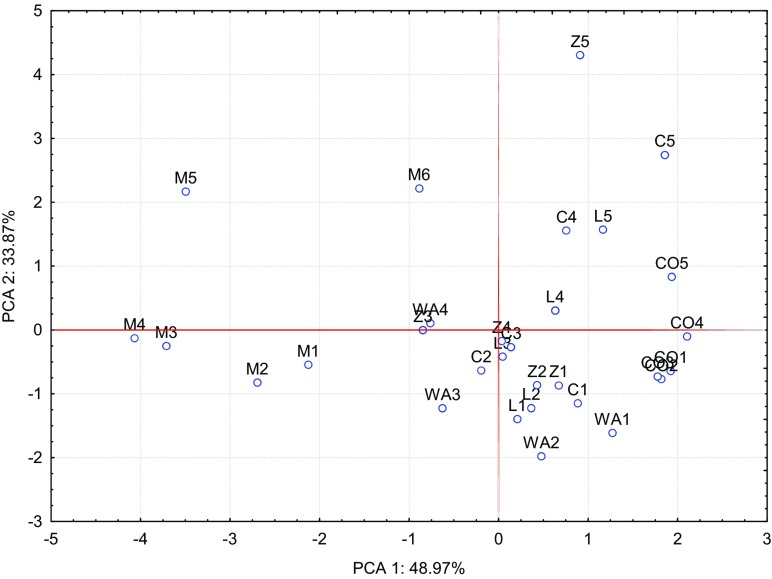
Effect of amendments on content of macroelements in the oats grain illustrated with the PCA method. Key: points show the grain samples with elements (*WA* without amendments, *M* manure, *L* clay, *C* charcoal, *Z* zeolite, *CO* calcium oxide, 1–0 mg, 2–20 mg, 3–40 mg, 4–80 mg, 5–160 mg, 6–320 mg Co/kg of soil)

**Fig. 3:**
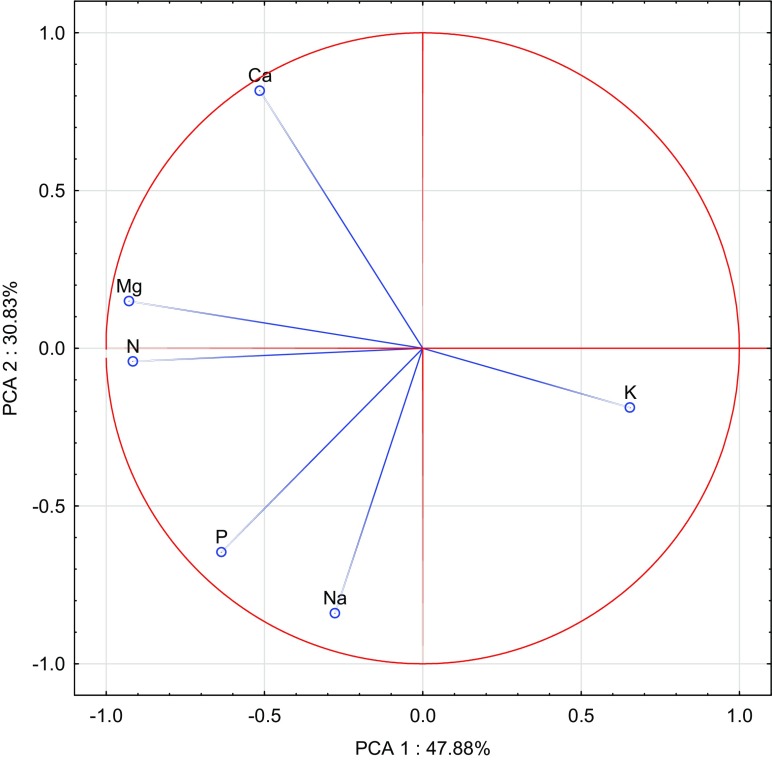
Content of macroelements in the oats straw illustrated with the PCA method. Key: vectors represent analysed variable (content of N, P, K, Na, Ca and Mg) and points show the straw samples with elements

**Fig. 4:**
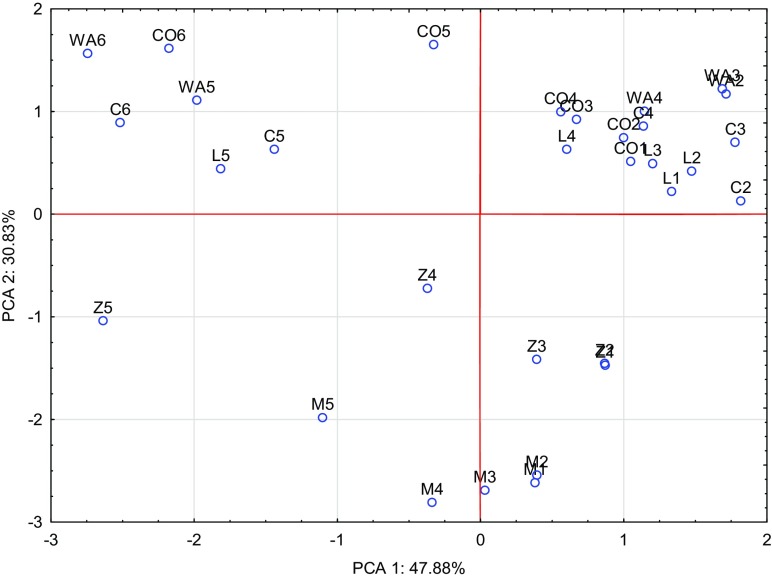
Effect of amendments on content of macroelements in the straw illustrated with the PCA method. Key: points show the oats straw samples with elements (*WA* without amendments, *M* manure, *L* clay, *C* charcoal, *Z* zeolite, *CO* calcium oxide, 1–0 mg, 2–20 mg, 3–40 mg, 4–80 mg, 5–160 mg, 6–320 mg Co/kg of soil)

**Fig. 5:**
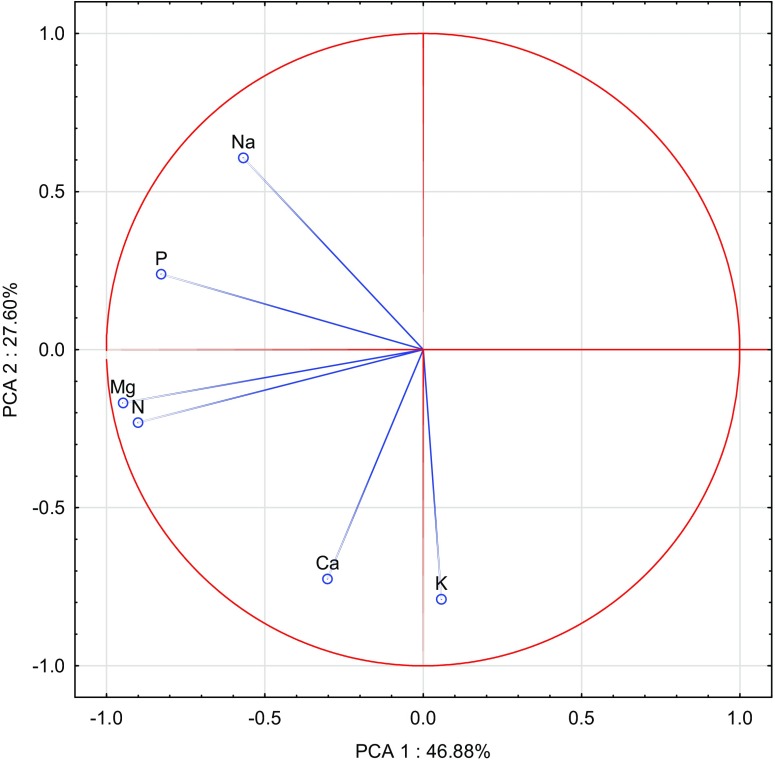
Content of macroelements in the oats roots illustrated with the PCA method. Key: vectors represent analysed variable (content of N, P, K, Na, Ca and Mg) and points show the roots samples with elements

**Fig. 6:**
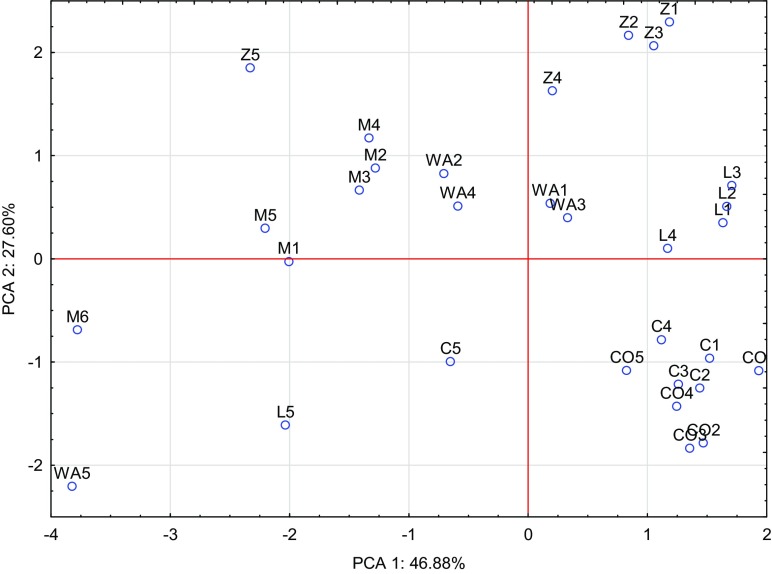
Effect of amendments on content of macroelements in the oats roots illustrated with the PCA method. Key: points show the roots samples with elements (*WA* without amendments, *M* manure, *L* clay, *C* charcoal, *Z* zeolite, *CO* calcium oxide, 1–0 mg, 2–20 mg, 3–40 mg, 4–80 mg, 5–160 mg, 6–320 mg Co/kg of soil)

The calculated correlation coefficients (Table 7) and results of the PCA performed (Figs. 1, 2, 3, 4, 5, and 6) confirm the presence of significant correlations between the content of particular macronutrients in the analysed organs of oat. PCA demonstrated changes in the content of macronutrients in oat grain under the influence of the applied cobalt doses and soil amendments (Fig. 1). The first group of macronutrients, which include potassium, phosphorus and sodium, constituted 48.97%, while the second group, composed of nitrogen, calcium and magnesium, corresponded to 33.87% of total correlation of the data set. Vectors of all analysed macronutrients were similar in length, hence justifying the claim that they had a significant contribution to the variance proportion. The strongest positive correlation was noted between sodium and potassium as well as between potassium and phosphorus, while the strongest negative correlation appeared between nitrogen and calcium. The dispersion of cases in the PCA evaluation, illustrated in Fig. 2, shows that the incorporation of manure and calcium oxide into soil had the strongest influence on the content of the analysed macronutrients in oat grain.

**Table 7:** Correlation coefficients (*r*) between content of macroelements in oats

	P	K	Na	Ca	Mg
Grain					
N	0.549**	0.259*	0.063	− 0.707**	− 0.187
P		0.620**	0.464**	− 0.510**	0.321**
K			0.695**	− 0.265*	0.499**
Na				− 0.047	0.711**
Ca					0.352**
Straw					
N	0.657**	− 0.392**	0.168	0.417**	0.786**
P		− 0.071	0.519**	− 0.186	0.502**
K			− 0.285**	− 0.398**	− 0.490**
Na				− 0.501**	0.079
Ca					0.564**
Roots					
N	0.617**	0.136	0.322**	0.309**	0.836**
P		− 0.114	0.443**	− 0.130	0.747**
K			− 0.452**	0.278*	− 0.050
Na				− 0.153	0.306**
Ca					0.415**

Significant at **P = 0.01, *P = 0.05

As regards oat straw, the first group of macronutrients (nitrogen, magnesium, potassium) constituted 47.88%, while the second group of macronutrients (sodium, phosphorus, calcium) composed 30.83% of total correlation of the data set (Fig. 3). Among vectors of all analysed macronutrients, the potassium vector was the shortest, implicating that this element had the smallest contribution to the variance proportion. Strong positive correlation was found between nitrogen and magnesium as well as phosphorus and sodium, while strong negative correlation appeared between potassium versus magnesium and nitrogen. Similarly to observations on oat grain, the strongest impact on the content of the analysed macronutrients in oat straw was produced by calcium oxide and manure added to soil, which is best illustrated by Fig. 4.

The principal component analysis results plotted in Fig. 5 reveal the content of macronutrients in oat roots after the soil application of increasing doses of cobalt and substances alleviating the effect of this metal. Magnesium, nitrogen and phosphorus represent 46.88% of correlation of the data set, while potassium, calcium and sodium correspond to 27.80%. The vectors of the macronutrients were of similar length, except magnesium and nitrogen, whose vectors were slightly longer than the others. Hence, it can be concluded that the contribution of these two elements to the building of principal components was the most significant. Strong positive correlation was determined between nitrogen and magnesium, and a weaker one between potassium and calcium as well as between phosphorus and sodium. Weak negative correlation was noted between potassium and sodium. The dispersion of PCA cases shown in Fig. 6 suggests that manure, zeolite and calcium oxide had the strongest effect on the content of macronutrients in roots of oat.

## Discussion

Cobalt belongs to elements which have a beneficial influence on the development of plants (Nessim and Abdalla 2008). Plants tend to accumulate small amounts of this element (Gál et al. 2008). According to Macnicol and Beckett (1985), the content of cobalt in plants within the range of 30–40 mg/kg can induce toxicity symptoms. The presence of high soil concentrations of cobalt can lead to the retardation of the growth of both roots and shoots of plants due to the cell division being slower, prolonged or both (Hemantaranjan et al. 2000; Wyszkowski et al. 2009). This is what was confirmed in our study, where the growth of oat in soil with the highest doses of cobalt was halted, thus preventing any analysis of plant material with regard to the content of the analysed macronutrients. When the development of plants becomes disrupted by high cobalt concentrations, the uptake of water and nutrients from soil by plants is depressed (Hemantaranjan et al. 2000). According to Vijayarengan and Dhanavel (2005), excess of trace elements in soil may affect the nutritional status of plants and cause interactions between some elements. The effect of high doses of cobalt, mostly inducing an increase in the content of nitrogen, phosphorus, sodium and calcium in all analysed parts of oat as well as potassium in grain and roots of this plant in the series without neutralising substances was also confirmed in this study. As the International Plant Nutrition Institute reports (2015b), the presence of cobalt in soil is beneficial to the uptake of nitrogen by plant roots, which was also noted in our research. Mocquot et al. (1996) concluded that the occurrence of excessively high concentrations of metals in soil significantly limited the transport of NO_3_ from roots of maize to higher organs of this plant. Nessim and Abdalla (2008) indicate that as a dose of cobalt in soil increased from 0 to 250 mg Co/kg, the content of phosphorus and potassium in spring barley decreased, and this relationship was most evident when higher cobalt doses had been applied to soil. The influence of cobalt applied in doses from 0 to 120 mg/kg of soil such as decreased content of potassium (by 15%) and increased content of phosphorus (by 19%), sodium (by 25%), magnesium (by 47%) and calcium (by 372%) in the aerial organs of oat had been demonstrated previously by Wyszkowski et al. (2009). This dependence was also shown in the current study, except for magnesium, whose content of in grain, straw or roots of the test plant was unaffected by the increasing doses of cobalt.

Toxic impact of cobalt on plants depends on many other factors apart from the content of this metal in soil. The ones most often mentioned are the soil reaction, content of organic matter, which enables complexing or immobilisation of cobalt, or the presence of other macronutrients and micronutrients in soil (Nessim and Abdalla 2008). Results of field experiments conducted by Rastija et al. (2014) indicate that the liming of acid soil in central Croatia with dolimite meal containing 56% CaO and 40% MgO and applied in doses of 5, 10 and 15 t/ha had a beneficial effect on soil pH, raising it to 6.36, 7.00 and 7.32, respectively. Moreover, the highest doses of dolomite led to an increase in the content of available forms of phosphorus by 8% in soil characterised by its high accumulation and by 45% in soil poor in this element.

In soil with alkaline reaction, the toxic effect of cobalt on plant development is weaker, mainly because of its intensive uptake by the sorption complex (Gál et al. 2008). The content of micronutrients in plants is also dependent on the presence of other elements in soil (Brodowska and Kaczor 2009). The analysis of our results clearly demonstrates that manure and calcium oxide had the strongest effect on the content of nitrogen, phosphorus, potassium, sodium and calcium in all organs of oat. It can therefore be concluded that these substances not only had a beneficial effect on the supply of plants with nutrients, but also most effectively mollified the harmful influence of high cobalt doses on the development of the test plant.

According to Silva et al. (2004), soil fertilisation with bovine manure applied in the cultivation of two maize cultivars had a substantial effect on the development of seed embryos as well as on the efficiency of seeds, increased water uptake and availability of phosphorus, potassium and sodium to plants in the soil layer to the depth of 20 cm (Silva et al. 2004). The International Plant Nutrition Institute (2015a) posits that the solubility of calcium is mostly dependent on the soil reaction. The higher the soil pH, the greater the solubility of calcium, as a result of which calcium becomes more phytoavailable. Moreover, incorporation of calcium to soil leads to the replacement of Na^+^ ions. As Mengel and Kirkby (2001) state, maintaining a proper level of potassium in plants improves the plant resistance owing to stronger cell walls. Compost and zeolite may contribute to a higher content of nitrogen in spring oilseed rape and in oat (Wyszkowski and Ziółkowska 2009). According to Ciećko et al. (2004, 2005), compost can raise the content of magnesium but decrease the content of potassium in most organs of analysed plants. In the experiment completed by Wyszkowski and Modrzewska (2015), zeolite had a more positive effect than compost and bentonite, as it led to a higher content of phosphorus, magnesium and calcium in plants. The study conducted by Sivitskaya and Wyszkowski (2013) showed that compost and zeolite caused a decrease in the content of phosphorus and an increase in the content of potassium in aerial parts of maize. In addition, compost led to an increase in the content of calcium, while zeolite elevated the content of calcium and magnesium in maize. In this study, the application of zeolite effected to a greater degree a decrease in the content of potassium in straw and roots of oat. The fact that zeolite, in the form of clinoptilolite, decreased the content of potassium in the test plant can be explained by its strong ion-exchange and adsorption properties towards some micro- and macronutrients present in soil (Manolov et al. 2005).

## Conclusion

The doses of cobalt applied in this experiment as well as soil amendments such as manure, clay, charcoal, zeolite and calcium oxide had a significant effect on the content of the analysed macronutrients in grain, straw and roots of oat. In the series without any neutralising substances, the soil contamination with cobalt caused an increase in the content of nitrogen, phosphorus, sodium, calcium and, partly, potassium, in grain, straw and roots of oat. Among the neutralising substances tested, the most unambiguous effect was produced by manure, which raised the content of all macronutrients (except calcium and magnesium) in oat grain, straw and roots. The influence of the other substances on the content of macronutrients in oat plants was less equivocal. However, all of them, especially calcium oxide, tended to induce a decrease in the content of most macronutrients in grain, straw and roots of oat.
